# Association of Nonsteroidal Anti-inflammatory Drug Use and Adverse Outcomes Among Patients Hospitalized With Influenza

**DOI:** 10.1001/jamanetworkopen.2020.13880

**Published:** 2020-07-01

**Authors:** Lars Christian Lund, Mette Reilev, Jesper Hallas, Kasper Bruun Kristensen, Reimar Wernich Thomsen, Christian Fynbo Christiansen, Henrik Toft Sørensen, Nanna Borup Johansen, Nikolai Constantin Brun, Marianne Voldstedlund, Henrik Støvring, Marianne Kragh Thomsen, Steffen Christensen, Anton Pottegård

**Affiliations:** 1 Clinical Pharmacology and Pharmacy, Department of Public Health, University of Southern Denmark, Odense, Denmark; 2 Department of Clinical Biochemistry and Clinical Pharmacology, Odense University Hospital, Odense, Denmark; 3 Department of Clinical Epidemiology, Aarhus University Hospital, Aarhus, Denmark; 4 Center for Population Health and Sciences, Stanford University, Stanford, California; 5 Department of Medical Evaluation and Biostatistics, Danish Medicines Agency, Copenhagen, Denmark; 6 Statens Serum Institut, Copenhagen, Denmark; 7 Department of Public Health–Biostatistics, Aarhus University, Aarhus, Denmark; 8 Department of Clinical Microbiology, Aarhus University Hospital, Aarhus, Denmark; 9 Department of Anaesthesia and Intensive Care Medicine, Aarhus University Hospital, Aarhus, Denmark; 10 Hospital Pharmacy Funen, Odense University Hospital, Odense, Denmark

## Abstract

**Importance:**

During the ongoing coronavirus disease 2019 pandemic, case reports have suggested that the use of nonsteroidal anti-inflammatory drugs (NSAIDs) may lead to adverse outcomes.

**Objective:**

To study the association of NSAID use with adverse outcomes in patients hospitalized with influenza or influenza pneumonia.

**Design, Setting, and Participants:**

This cohort study used propensity score matching among 7747 individuals aged 40 years or older who were hospitalized with influenza, confirmed by polymerase chain reaction or antigen testing, between 2010 and 2018. Data were collected using Danish nationwide registers. All analyses reported were performed on May 29, 2020.

**Exposures:**

Prescription fill of an NSAID within 60 days before admission.

**Main Outcomes and Measures:**

Risk ratio (RR) and risk difference (RD) with 95% CIs for intensive care unit admission and death within 30 days of admission.

**Results:**

A total of 7747 patients (median [interquartile range] age, 71 [59-80] years, 3980 [51.4%] men) with confirmed influenza were identified. Of these, 520 (6.7%) were exposed to NSAIDs. In the unmatched cohorts, 104 of 520 patients (20.0%) who used NSAIDs and 958 of 7227 patients (13.3%) who did not use NSAIDs were admitted to the intensive care unit. For death within 30 days of admission, we observed 37 events (7.1%) among those who used NSAIDs compared with 563 events (7.8%) among those who did not. Current NSAID use was associated with intensive care unit admission (RR, 1.51; 95% CI, 1.26 to 1.81; RD, 6.7%; 95% CI, 3.2% to 10.3%), while NSAID use was not associated with death (RR, 0.91; 95% CI, 0.66 to 1.26; RD, –0.7%; 95% CI, –3.0% to 1.6%). In the matched cohorts, risks were unchanged for patients who used NSAIDs, while 83 ICU admissions (16.0%) and 36 deaths (6.9%) were observed among matched individuals who did not use NSAIDs. Matched (ie, adjusted) analyses yielded attenuated risk estimates for intensive care unit admission (RR, 1.25; 95% CI, 0.95 to 1.63; RD, 4.0%; 95% CI, –0.6% to 8.7%) and death (RR, 1.03; 95% CI, 0.66 to 1.60; RD, 0.2%; 95% CI, –2.9% to 3.3%). Associations were more pronounced among patients who used NSAIDs for a longer period (eg, for intensive care unit admission: RR, 1.90; 95% CI, 1.19 to 3.06; RD, 13.4%; 95% CI, 4.0% to 22.8%).

**Conclusions and Relevance:**

In this cohort study of adult patients hospitalized with influenza, the use of NSAIDs was not associated with 30-day intensive care unit admission or death in adjusted analyses. There was an association between long-term use of NSAIDs and intensive care unit admission.

## Key Points

**Question:**

Is exposure to nonsteroidal anti-inflammatory drugs (NSAIDs) associated with increased risk of intensive care unit admission or death among patients hospitalized with influenza?

**Findings:**

In this Danish nationwide cohort study that included 7747 adults aged 40 years or older, no association of NSAID use with intensive care unit admission or 30-day mortality was found.

**Meaning:**

The findings of this study suggest that among patients hospitalized with influenza, those who use NSAIDs do not have worse prognosis than those with no NSAID use.

## Teaser

This cohort study examines the association of nonsteroidal anti-inflammatory drugs (NSAIDs) with adverse outcomes in patients hospitalized with influenza or influenza pneumonia.

## Introduction

Exposure to nonsteroidal anti-inflammatory drugs (NSAIDs) has been associated with an increased occurrence of pleuropulmonary complications, such as empyema, in patients with bacterial pneumonia.^1,2^ During the current coronavirus disease 2019 (COVID-19) pandemic, case reports have described patients with no comorbidities who developed severe COVID-19 after using NSAIDs in the early stage of the disease.^3^ While the European Medicines Agency stated that there is currently no scientific evidence establishing an association between use of NSAIDs and the severity of COVID-19,^4^ uncertainty persists regarding whether NSAID use can be considered safe in this disease.^3^

Existing evidence of the association between NSAID use and adverse outcomes in patients with pneumonia is based on patients with bacterial or unspecified pneumonia,^1,2,5^ with the most commonly reported outcome of pleuropulmonary complications (empyema or abscesses) having little relevance in patients with influenza or COVID-19. Explanations other than a direct drug effect have also been proposed for adverse outcomes among those receiving NSAIDs.^1^ They primarily concern the risk of confounding by indication, ie, that early symptoms of complications may lead to the use of NSAIDs, thereby introducing a spurious association between NSAIDs and adverse outcomes. Further, use of NSAIDs may mask pneumonia symptoms because of their analgesic and antipyretic effects, thus leading to delayed health care seeking and delayed antibiotic prescriptions for secondary bacterial infections,^6^ which can in turn lead to more severe disease and prolonged hospital stays.^1,2^

Given the widespread use of NSAIDs and the rapidly evolving COVID-19 pandemic, studies are urgently needed to determine the association of NSAIDs with the prognosis of viral pneumonia. Therefore, we examined the association between NSAID use and adverse outcomes following hospitalization for influenza or influenza-related pneumonia.

## Methods

In this Danish registry-based cohort study covering all Danish hospitals, we constructed cohorts of patients hospitalized for influenza or pneumonia from 2010 to 2018 and investigated the association between current NSAID use (ie, preadmission NSAID use) and risk of adverse outcomes, specified as an intensive care unit (ICU) stay, pleuropulmonary complications (empyema or pulmonary abscess), and death. The study was approved by the institutional data protection board at the University of Southern Denmark and the Danish Health Data Authority. According to Danish law, studies based entirely on registry data do not require approval from an ethics review board or informed consent.^7^ Because of Danish privacy regulations, counts of less than 5 cannot be reported. Therefore, absolute numbers are only reported for the most important risk estimates. This study follows the Strengthening the Reporting of Observational Studies in Epidemiology (STROBE) reporting guideline for cohort studies.

### Data Sources

Denmark has a tax-supported health care system that provides free access to health care. We retrieved data from 4 nationwide Danish health registries: the National Prescription Registry,^8^ the Danish National Patient Registry,^9^ the Danish Civil Registration System,^10^ and the Danish Microbiology Database.^11^ Data were linked by using unique personal registration numbers, which are provided to all Danish residents.^10^

The National Prescription Registry records data on all prescriptions redeemed by Danish residents at outpatient pharmacies from 1995 to the present.^8^ Among other variables, prescription data include the date of dispensing and the substance dispensed, categorized according to the Anatomical Therapeutic Chemical classification. The Danish National Patient Registry contains data on all hospital admissions since 1977 and all contacts with outpatient clinics since 1995. Data include information on date of admission and discharge diagnoses classified according to the *International Classification of Diseases, Tenth Revision* (*ICD*-*10*).^9^ The Danish Civil Registration System covers every Danish resident and records data on vital status (date of birth and death) and migration to and from Denmark.^10^ The Danish Microbiology Database contains nationwide information on microbiological test results starting in 2010, including results for polymerase chain reaction and antigen tests for the influenza virus.^10^ Detailed information on codes used to define cohorts, exposures, and outcomes are provided in eAppendix 1 in the Supplement.

### Study Population

We included all Danish patients aged 40 years and older who were hospitalized with an *ICD*-*10* code related to pneumonia or influenza from 2010 to 2018 (N = 563 522). Individuals with less than 1 year of enrollment in the database before admission or who emigrated within 30 days of admission were excluded from the analysis (n = 325).

From the pool of eligible patients, we identified several study cohorts. For the main analysis, we restricted the cohort to patients with first-time hospital admission because of confirmed influenza or influenza-related pneumonia. This was defined as a discharge diagnosis of any type of influenza or pneumonia, in combination with a positive polymerase chain reaction or antigen test for influenza virus from 14 days before admission to discharge or 30 days after admission, whichever occurred first. In secondary analyses, we analyzed cohorts comprised of patients with a first-time hospital admission because of any bacterial pneumonia or *Streptococcus pneumoniae* pneumonia, except for unspecified pneumonia codes. The secondary cohorts were defined based on diagnosis codes alone. For all cohorts, patients entered the cohort on the date of hospital admission and were observed for 30 days or until they experienced an outcome of interest.

### Drug Exposure

The main exposure of interest was current use of NSAIDs. Current use was defined as having filled at least 1 prescription for any NSAID within 60 days before hospital admission. In secondary analyses, we also examined incident use and long-term use of NSAIDs, which provided information on different patient groups and also allowed for the assessment of potential confounding by indication. Incident use was defined as a first redemption of an NSAID in the 14 days before hospital admission following a washout period of 1 year. Long-term use was defined as a subset of current use, with the additional requirement of having redeemed an average of 0.5 defined daily doses of NSAIDs per day (corresponding to, eg, 600 mg ibuprofen or 50 mg diclofenac per day) during the previous year. In the main analysis, current, incident, and long-term use were all compared with nonuse of NSAIDs. In additional analyses, we used acetaminophen use as an active comparator. Acetaminophen use was defined according to the same criteria as NSAID use, so that incident use of NSAIDs was comparable with incident use of acetaminophen.

Use of NSAIDs was determined using data on prescription fills.^8^ In Denmark, only low-dose ibuprofen (ie, 200 mg) can be obtained over the counter and is not captured in the registry data. During the study period, over-the-counter sales of ibuprofen accounted for 27.2% of total ibuprofen sales during the study period (eAppendix 2 in the Supplement).^12^ Furthermore, over-the-counter use is expected to be less frequent among older individuals, who are eligible for considerable reimbursement for the cost of prescription drugs.^13^

### Outcomes

The outcomes of interest were admission to an ICU within 30 days of hospital admission and death within 30 days of hospital admission. For patients with bacterial pneumonia, pleuropulmonary complications were an additional outcome.

### Propensity Score Matching

We used propensity score (PS) matching^14^ to adjust for observed differences between the treated and untreated cohorts. A PS value is the estimated probability of treatment given a set of patient characteristics. Each individual’s PS was estimated at cohort entry based on known confounders and risk factors for adverse outcomes. These included age (as a continuous variable), sex, year of cohort entry, prior use of selected prescription drugs, and comorbidities. The full list of included covariates is provided in eAppendix 1 in the Supplement. Separate PSs were estimated for all combinations of subcohorts, choice of comparator cohort, and exposure definitions (n = 18). To reduce unmeasured confounding, we performed asymmetrical trimming of the PS distribution^15^ before matching, ie, we removed patients who were treated contrary to prediction to the greatest extent. Individuals in the untreated cohort were matched to individuals in the treated cohort in a 1:1 ratio using the nearest neighbor algorithm without replacement and a maximum caliper of 0.05.

### Statistical Analysis

Descriptive statistics were used to characterize the study cohorts at baseline. In both the unmatched and matched cohorts, we estimated risks and absolute risk differences (RDs) with generalized linear models using a binomial distribution and an identity link. Risk ratios (RRs) were estimated using generalized linear models with a binomial distribution and a log link, and 95% CIs were obtained. To assess whether risk estimates were modified by age (ie, <65 and ≥65 years), sex, and cardiovascular disease (an established relative contraindication for NSAID use^16^), we included these factors as interaction terms in the generalized linear models. Likelihood ratio tests were used to test for interaction.

We performed a number of supplementary and sensitivity analyses. First, given that ibuprofen specifically has been implied to worsen the prognosis of patients with pneumonia or COVID-19,^3^ we repeated the main analysis with ibuprofen use defined as the exposure. Second, we performed analyses restricting the 30-day mortality outcome to in-hospital deaths only, ie, censoring patients at discharge. Third, to assess the robustness of our findings toward choice of PS methodology, we repeated the main analyses using inverse probability of treatment weights^17^ to address differences between treatment groups. This also changed the underlying causal contrast from the average treatment effect among those treated when matching to the average treatment effect in the population. Fourth, we obtained risk estimates for individuals with community-acquired influenza only, ie, excluding individuals with a positive test more than 2 days after the initial hospital admission.

All analyses reported were performed on May 29, 2020. Statistical analyses were conducted using Stata MP version 16.1 (StataCorp). Statistical significance was set at *P* < .05, and all tests were 2-tailed.

## Results

We identified 7747 patients with confirmed influenza or influenza-related pneumonia between January 1, 2010, and December 31, 2018 (Figure). The median (interquartile range [IQR]) age was 71 (59-80) years, and 3980 patients (51.4%) were men. A total of 6678 individuals (86.2%) had their positive polymerase chain reaction or antigen test for influenza within 3 days before or after admission. Overall, the 30-day risk of death was 7.7% (600 of 7747 individuals), while the risk of ICU admission was 13.7% (1062 of 7747 individuals). In total, 520 patients (6.7%) were currently using NSAIDs at the time of hospital admission. These individuals were younger than individuals who did not use NSAIDs (median (IQR) age, 66 [54-74] years vs 71 [60-80] years) and a higher proportion used nonbenzodiazepine benzodiazepine receptor agonists (80 [15.4%] vs 717 [9.9%]) and had received antibiotics before admission (159 [30.6%] vs 1680 [23.2%]). Compared with those who did not use NSAIDs, fewer individuals using NSAIDs had cardiovascular disease (2860 [39.6%] vs 146 [28.1%]) or used low-dose aspirin (1752 [24.2%] vs 101 [19.4%]) (Table 1).

**Figure.**
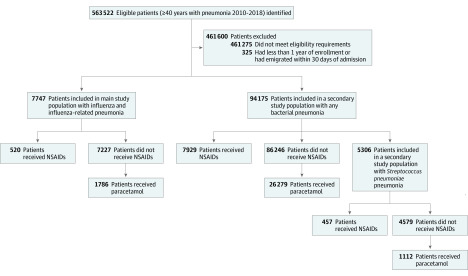
Identification of Study Population NSAID indicates nonsteroidal anti-inflammatory drug.

**Table 1.** Baseline Characteristics of the Main Study Cohort, Before and After Propensity Score Matching of Treatment Groups

Characteristic	Total (N = 7747)	Unmatched			Matched		
		Using NSAIDs (n = 520)	Not using NSAIDs (n = 7227)	SMD	Using NSAIDs (n = 520)	Not using NSAIDs (n = 520)	SMD
Age, median (IQR), y	71 (59-80)	66 (54-74)	71 (60-80)	0.36	66 (54-74)	64 (55-75)	0.02
Men	3980 (51.4)	232 (44.6)	3748 (51.9)	0.15	232 (44.6)	238 (45.8)	0.02
Current drug use							
Antihypertensives	5600 (72.3)	363 (69.8)	5237 (72.5)	0.06	363 (69.8)	343 (66.0)	0.08
Antidiabetics	1503 (19.4)	102 (19.6)	1401 (19.4)	0.01	102 (19.6)	104 (20.0)	0.01
Low-dose aspirin	1853 (23.9)	101 (19.4)	1752 (24.2)	0.12	101 (19.4)	101 (19.4)	0.00
Immunosuppressants	173 (2.2)	14 (2.7)	159 (2.2)	0.03	14 (2.7)	16 (3.1)	0.02
Recent antibiotics	1839 (23.7)	159 (30.6)	1680 (23.2)	0.17	159 (30.6)	142 (27.3)	0.07
Monotherapy β-lactams	1039 (13.4)	78 (15.0)	961 (13.3)	0.05	78 (15.0)	64 (12.3)	0.08
β-lactams combined with β-lactamase inhibitors	404 (5.2)	41 (7.9)	363 (5.0)	0.12	41 (7.9)	38 (7.3)	0.02
Macrolides	383 (4.9)	39 (7.5)	344 (4.8)	0.11	39 (7.5)	39 (7.5)	0.00
Quinolones	81 (1.0)	5 (1.0)	76 (1.1)	0.01	5 (1.0)	<5 (NA)	NA
Opioids	2179 (28.1)	232 (44.6)	1947 (26.9)	0.38	232 (44.6)	224 (43.1)	0.03
Nonbenzodiazepine benzodiazepine receptor agonists	797 (10.3)	80 (15.4)	717 (9.9)	0.16	80 (15.4)	78 (15.0)	0.01
Benzodiazepines	997 (12.9)	79 (15.2)	918 (12.7)	0.07	79 (15.2)	63 (12.1)	0.09
First-generation antipsychotics	204 (2.6)	27 (5.2)	177 (2.4)	0.14	27 (5.2)	32 (6.2)	0.04
Second-generation antipsychotics	330 (4.3)	29 (5.6)	301 (4.2)	0.07	29 (5.6)	21 (4.0)	0.07
Systemic glucocorticoids	1042 (13.5)	98 (18.8)	944 (13.1)	0.16	98 (18.8)	91 (17.5)	0.03
Inhaled corticosteroids	1361 (17.6)	115 (22.1)	1246 (17.2)	0.12	115 (22.1)	117 (22.5)	0.01
Comorbidities							
Asthma	816 (10.5)	64 (12.3)	752 (10.4)	0.06	64 (12.3)	62 (11.9)	0.01
COPD	1709 (22.1)	113 (21.7)	1596 (22.1)	0.01	113 (21.7)	114 (21.9)	0.00
Cardiovascular disease	3006 (38.8)	146 (28.1)	2860 (39.6)	0.24	146 (28.1)	133 (25.6)	0.06
Ischaemic stroke	865 (11.2)	51 (9.8)	814 (11.3)	0.05	51 (9.8)	52 (10.0)	0.01
Chronic kidney failure	323 (4.2)	17 (3.3)	306 (4.2)	0.05	17 (3.3)	20 (3.8)	0.03
Liver disease	258 (3.3)	20 (3.8)	238 (3.3)	0.03	20 (3.8)	25 (4.8)	0.05
Alcohol-related disorders	549 (7.1)	40 (7.7)	509 (7.0)	0.02	40 (7.7)	40 (7.7)	0.00
Dementia	250 (3.2)	8 (1.5)	242 (3.3)	0.12	8 (1.5)	9 (1.7)	0.02
Cancer	1779 (23.0)	117 (22.5)	1662 (23.0)	0.01	117 (22.5)	106 (20.4)	0.05
HIV	23 (0.3)	<5 (NA)	NA	NA	<5 (NA)	NA	NA
Obesity	631 (8.1)	47 (9.0)	584 (8.1)	0.03	47 (9.0)	45 (8.7)	0.01
Hemiplegia and paraplegia	103 (1.3)	6 (1.2)	97 (1.3)	0.02	6 (1.2)	8 (1.5)	0.03

Abbreviations: COPD, chronic obstructive pulmonary disease; IQR, interquartile range; NA, not applicable; NSAID, nonsteroidal anti-inflammatory drug; SMD, standardized mean difference.

In the unmatched cohorts with confirmed influenza or influenza-related pneumonia, 104 patients (20.0%) who used NSAIDs and 958 patients (13.3%) who did not were admitted to the ICU, yielding an RR of 1.51 (95% CI, 1.26 to 1.81) and an RD of 6.7% (95% CI, 3.2% to 10.3%). Of those who used NSAIDs, 37 (7.1%) died within 30 days of admission, compared with 563 (7.8%) of those who did not use NSAIDs, resulting in an RR of 0.91 (95% CI, 0.66 to 1.26) and a RD of –0.7% (95% CI, –3.0% to 1.6%) (eTable 1 in the Supplement).

After PS matching (eTable 2 in the Supplement), the standardized mean difference was less than 0.1 for all baseline covariates, indicating successful balancing of measured confounders (Table 1). PS-matched analyses were generally similar to the unmatched analyses but with slightly attenuated risk estimates for use of NSAIDs. Risks of ICU admission and death were unchanged among matched patients who were currently using NSAIDs (20.0% [95% CI, 16.6% to 23.4%] and 7.1% [95% CI, 4.9% to 9.3%], respectively). Among matched patients who were not currently using NSAIDs, 83 individuals (16.0%; 95% CI, 12.8% to 19.1%) were admitted to the ICU, yielding an RR of 1.25 (95% CI, 0.96 to 1.63) and an RD of 4.0% (95% CI, –0.6% to 8.7%). For death within 30 days of admission, 36 events (6.9%; 95% CI, 4.7% to 9.1%) were observed among matched patients who were not using NSAIDs, resulting in an RR of 1.03 (95% CI, 0.66 to 1.60) and an RD of 0.2% (95% CI, –2.9 to 3.3%) (Table 2). Restricting exposure to current use of ibuprofen did not change the results (eTable 3 in the Supplement). In analyses restricted to incident use of NSAIDs with confirmed influenza, the RRs of ICU admission and death were 1.40 (95% CI, 0.68 to 2.88) and 1.00 (95% CI, 0.26 to 3.80), respectively, corresponding to RDs of 7.1% (95% CI, –8.0% to 22.3%) and 0.0% (95% CI, –9.5 to 9.5%), respectively. Similarly, in analyses restricted to long-term NSAID use, the RRs of ICU admission and death were 1.90 (95% CI, 1.19 to 3.06) and 1.43 (95% CI, 0.56 to 3.65), respectively, corresponding to RDs of 13.4% (95% CI, 4.0% to 22.8%) and 2.1% (95% CI, –3.4% to 7.6%), respectively (Table 2). We observed that RRs were elevated among those younger than 65 years of age, women, and those without a history of cardiovascular disease (eTable 4 in the Supplement), although these differences achieved statistical significance only for the comparison of men and women (RR for ICU admission among men, 0.89; 95% CI, 0.60 to 1.31; RR for ICU admission among women, 1.67; 95% CI, 1.16 to 2.41; *P* = .02). Restricting 30-day mortality to in-hospital death led to estimates similar to those obtained in the main analysis (eTable 5 in the Supplement). Replication of the main analysis using inverse probability of treatment weights yielded similar results (eTable 6 in the Supplement). When restricting the inclusion criteria to individuals with community-acquired influenza, current use of NSAIDs was not associated with ICU admission (RR, 1.17; 95% CI, 0.85 to 1.62; RD, 2.3%; 95% CI, –2.4% to 7.1%) or 30-day mortality (RR, 0.71; 95% CI, 0.39 to 1.30; RD, –1.6%; 95% CI, –4.5% to 1.2%), as in the main analysis.

**Table 2.** Associations Between Current, Incident, and Long-term Use of NSAIDs in the Main Propensity-Matched Study Cohort

Exposure	Risk among NSAID use cohort, % (95% CI)	Risk among NSAID nonuse cohort, % (95% CI)	Risk difference, % (95% CI)	Risk ratio (95% CI)
Intensive care unit admission				
Current use	20.0 (16.6 to 23.4)	16.0 (12.8 to 19.1)	4.0 (–0.6 to 8.7)	1.25 (0.96 to 1.63)
Incident use	25.0 (13.7 to 36.3)	17.9 (7.8 to 27.9)	7.1 (–8.0 to 22.3)	1.40 (0.68 to 2.88)
Long-term use	28.2 (20.8 to 35.6)	14.8 (9.0 to 20.6)	13.4 (4.0 to 22.8)	1.90 (1.19 to 3.06)
30-d mortality				
Current use	7.1 (4.9 to 9.3)	6.9 (4.7 to 9.1)	0.2 (–2.9 to 3.3)	1.03 (0.66 to 1.60)
Incident use	7.1 (0.4 to 13.9)	7.1 (0.4 to 13.9)	0.0 (–9.5 to 9.5)	1.00 (0.26 to 3.80)
Long-term use	7.0 (2.8 to 11.3)	4.9 (1.4 to 8.5)	2.1 (–3.4 to 7.6)	1.43 (0.56 to 3.65)

Abbreviation: NSAID, nonsteroidal anti-inflammatory drug.

When those who used acetaminophen were used as the active comparator for patients with influenza and influenza-related pneumonia, we generally observed larger differences in baseline characteristics than when using individuals who did not use NSAIDs as the comparator. Compared with those who used NSAIDs, patients who used acetaminophen were older (median [IQR] age, 66 [54-74] years vs 76 [67-83] years), more often used antihypertensive (363 of 520 [69.8%] vs 1508 of 1786 [84.4%]) and antidiabetic (102 [19.6%] vs 435 [24.4%]) medications, and had a higher prevalence of multiple chronic conditions (eg, chronic obstructive pulmonary disease, 113 [21.7%] vs 556 [31.1%]; cardiovascular disease, 146 [28.1%] vs 923 [51.7%]) (eTable 7 in the Supplement). Compared with risk estimates observed when those who did not use NSAIDs were comparators, we observed larger risk estimates in the unmatched analyses and a larger effect of matching. This indicates that, compared with those who did not use NSAIDs, those who used acetaminophen were less comparable with those who used NSAIDs. This suggests a higher risk of residual confounding. The matched RRs for ICU admission and death were 1.16 (95% CI, 0.89 to 1.53) and 0.83 (95% CI, 0.54 to 1.28), respectively, corresponding to RDs of 2.7% (95% CI, –2.1% to 7.5%) and –1.4% (95% CI, –4.9% to 2.0%), respectively.

Within the secondary cohort of individuals with bacterial pneumonia comparing NSAID use to nonuse, the matched RR of ICU admission was 1.15 (95% CI, 1.06 to 1.24), and the RD was 1.8% (95% CI, 0.8% to 2.9%). For death, an RR of 0.97 (95% CI, 0.90 to 1.05) and an RD of –0.4% (95% CI, –1.5% to 0.7%) were obtained. We observed 168 events (2.1%) and 79 events (1.0%) of pleuropulmonary complications in matched cohorts of NSAID use and nonuse, respectively, corresponding to an RR of 2.13 (95% CI, 1.63 to 2.77) and an RD of 1.1% (95% CI, 0.7% to 1.5%). Likewise, incident and long-term use of NSAIDs were also associated with pleuropulmonary complications, with RRs of 3.67 (95% CI, 1.95 to 6.91) and 1.87 (95% CI, 1.13 to 3.09), respectively, and RDs of 2.8% (95% CI, 1.5% to 4.1%) and 0.9% (95% CI, 0.2% to 1.6%), respectively (eTable 8 in the Supplement). Similar estimates were observed when restricting to patients with *Streptococcus pneumonia*e pneumonia (eTable 9 in the Supplement).

## Discussion

In this nationwide population-based cohort study of patients with confirmed influenza or influenza-related pneumonia, we found that NSAID use was not associated with the risk of 30-day mortality or ICU admission. Similar associations for ICU admission and death were observed for patients with bacterial pneumonia. In accordance with previous studies, we also observed an increased risk of pleuropulmonary complications for NSAID users with bacterial pneumonia.

A causal biological association between NSAID use and adverse pneumonia outcomes is plausible. NSAIDs impair neutrophil function and recruitment to the inflammatory site through cyclooxygenase-2 inhibition and may thus delay the resolution of inflammatory processes.^18^ In addition, NSAID use has been suggested to increase the risk of renal failure in critically ill patients^19^ and may also increase the risk of cardiovascular complications.^20^ Finally, NSAIDs may mask the earliest symptoms of an adverse outcome in pneumonia by relieving fever and pleural pain, potentially delaying medical assistance. This is indicated by the findings of Little et al,^5^ who observed that individuals who used ibuprofen had more revisits with their general practitioner than those who used acetaminophen (20% vs 12%) because of unresolved symptoms or complications.^5^

Evidence is limited concerning the safety of NSAIDs in patients with viral lower respiratory tract infections. Preadmission NSAID use was not associated with mortality in ICU patients with severe influenza H1N1 during the 2009 pandemic.^21^ However, the study’s restriction to patients already admitted to the ICU prohibits evaluation of whether NSAID use increased the risk of ICU admission in the first place. Other studies have reported paradoxically low mortality in older patients who used NSAIDs.^22^ A plausible interpretation is that NSAIDs are withheld from the frailest older patients because of the well-established detrimental effect of NSAIDs on the kidneys, heart, and gastrointestinal tract, hence conferring apparently low mortality among the remaining older patients who used NSAIDs.^22^ The positive association of NSAID use with increased risk of ICU admission may reflect the same phenomenon, ie, both NSAID therapy and ICU admission were less likely to be offered to the frailest individuals, among whom longer-term outcomes may be unfavorable. This possibility may be supported by the fact that NSAID use was associated with a reduced relative risk of ICU admission among older patients and patients with cardiovascular disease compared with younger patients and patients without cardiovascular disease (eTable 3 in the Supplement). We strived to eliminate confounding by using PS techniques, but we cannot rule out some residual confounding by unmeasured frailty.

The main body of literature on NSAIDs and the increased risk of adverse outcomes in patients with pneumonia focuses on pleuropulmonary complications, especially pleural empyema and lung abscesses, in bacterial or unspecified pneumonia.^1,6,23,24^ A recurrent problem in interpreting this literature is the possibility of confounding by indication or protopathic bias. The earliest stages of a pleural empyema may manifest as dry pleurisy, which is likely to be painful and treated with analgesics. This may manifest in the data as a strong association between recently started NSAID therapy and a diagnosis of pleural empyema. Indeed, for pleuropulmonary complications of bacterial pneumonias, we found the strongest association with recently started NSAID use (RR, 3.67; 95% CI, 1.95-6.91). However, there was also a clear association with long-term NSAID use (RR, 1.87; 95% CI, 1.13-3.09). This implies that confounding by indication does not explain this association in its entirety, and masking early symptoms (as discussed earlier) might also contribute to this finding.

The findings of this study should be interpreted in the context of a recent warning against using NSAIDs in patients suspected of having COVID-19 infection. The literature underlying this recommendation is largely based on cohorts of patients with unspecified or bacterial pneumonias and the risk of pleuropulmonary complications. Regarding coronavirus infections, NSAID use may specifically cause an upregulation of angiotensin-converting enzyme 2 receptors, similar to renin-angiotensin-aldosterone system blockers.^25^ Given that this receptor is used by coronaviruses to bind to epithelial cells of the lung and other organs,^26^ NSAIDs may specifically increase the risk of severe and fatal COVID-19. The same biological mechanism may not necessarily apply to the influenza cohort in our study, and therefore the null findings do not necessarily reject the existence of an association between NSAID use and a worse prognosis of COVID-19.

### Strengths and Limitations

The principle strength of our study is the use of data obtained from the Danish health registries, which are generally considered to be of high validity and provide nationwide coverage, thereby limiting the risk of selection bias. The main limitation is the limited granularity of data, which did not include, eg, direct measures of frailty. Also, PS methods are effective for removing measured confounding but do not generally adjust for unmeasured confounding. As such, some residual confounding cannot be ruled out. Furthermore, microbiological verification was only possible for the main cohort of patients with confirmed influenza or influenza-related pneumonia. Secondary cohorts of pneumonia were defined using diagnosis codes only. However, the positive predictive value of a hospital diagnosis of pneumonia in Denmark is high (90%).^27^ In addition, given that we restricted to influenza, inferences cannot be made for less common viral infections, such as varicella zoster pneumonia, for which the pathologies are markedly different.^28^ Also, we had no data on bacterial superinfections, which are a common complication among patients admitted because of influenza.^28^ Exposure to NSAIDs is known to be sporadic and difficult to model.^29^ Thus, some exposure misclassification may occur. However, because uncaptured NSAID use among those who were categorized as not using NSAIDs is expected to be uncommon, this is not expected to have any major influence on the study findings.

## Conclusions

In this study, NSAID use was not associated with a clinically significant increased risk of ICU admission or death in patients hospitalized with influenza. While studies on the association of NSAIDs with the disease course of COVID-19 are clearly needed, the currently available data, including the present study, do not seem to support strong recommendations against using NSAIDs in patients with viral pneumonia.

## Notes

Supplement. **eAppendix 1. **Codes Used to Define Eligibility Criteria, Exposures, Outcomes, and Covariates **eAppendix 2. **NSAID Sales in Denmark, Used for Calculation of Over-the-Counter NSAID Sales **eTable 1. **Risk, Risk Differences, and Risk Ratios for Main Cohort, Unmatched Cohorts **eTable 2. **Univariate Association with Death and Current Use of NSAIDs for Each Covariate Included in the Propensity Score **eTable 3. **Risks, Risk Differences, and Risk Ratios for Ibuprofen Use, Main Cohort **eTable 4. **Estimates of Risk Ratios When Investigating Potential Effect Modification by Age, Sex, and History of Cardiovascular Disease **eTable 5. **Risks, Risk Differences, and Risk Ratios for In-Hospital Death, Main Population **eTable 6. **Risks, Risk Differences, and Risk Ratios for Main Cohort Using Inverse Probability of Treatment Weights **eTable 7. **Baseline Characteristics of Those Currently Using NSAIDs and Those Currently Using Acetaminophen **eTable 8. **Risks, Risk Differences, and Risk Ratios for Cohort Diagnosed With any Bacterial Pneumonia **eTable 9. **Risks, Risk Differences, and Risk Ratios for Cohort Diagnosed With *Streptococcus pneumoniae *Pneumonia
