# Supplementary material for: Association of Nonsteroidal Anti-inflammatory Drug Use and Adverse Outcomes Among Patients Hospitalized With Influenza
Source: JAMA Netw Open. 2020 Jul 1;3(7):e2013880. doi: 10.1001/jamanetworkopen.2020.13880 (PMC7330719; doi:10.1001/jamanetworkopen.2020.13880)
Supplement: Supplement. — eAppendix 1. Codes Used to Define Eligibility Criteria, Exposures, Outcomes, and Covariates eAppendix 2. NSAID Sales in Denmark, Used for Calculation of Over-the-Counter NSAID Sales eTable 1. Risk, Risk Differences, and Risk Ratios for Main Cohort, Unmatched Cohorts eTable 2. Univariate Association with Death and Current Use of NSAIDs for Each Covariate Included in the Propensity Score eTable 3. Risks, Risk Differences, and Risk Ratios for Ibuprofen Use, Main Cohort eTable 4. Estimates of Risk Ratios When Investigating Potential Effect Modification by Age, Sex, and History of Cardiovascular Disease eTable 5. Risks, Risk Differences, and Risk Ratios for In-Hospital Death, Main Population eTable 6. Risks, Risk Differences, and Risk Ratios for Main Cohort Using Inverse Probability of Treatment Weights eTable 7. Baseline Characteristics of Those Currently Using NSAIDs and Those Currently Using Acetaminophen eTable 8. Risks, Risk Differences, and Risk Ratios for Cohort Diagnosed With any Bacterial Pneumonia eTable 9. Risks, Risk Differences, and Risk Ratios for Cohort Diagnosed With Streptococcus pneumoniae Pneumonia [file jamanetwopen-3-e2013880-s001.pdf]

Supplementary Online Content

Lund LC, Reilev M, Hallas J, et al. Association of nonsteroidal anti-inflammatory drug use and adverse outcomes among patients hospitalized with influenza. *JAMA Netw Open*. 2020;3(7):e2013880. doi:10.1001/jamanetworkopen.2020.13880

eAppendix 1. Codes Used to Define Eligibility Criteria, Exposures, Outcomes, and Covariates

eAppendix 2. NSAID Sales in Denmark, Used for Calculation of Over-the-Counter NSAID Sales

eTable 1. Risk, Risk Differences, and Risk Ratios for Main Cohort, Unmatched Cohorts

eTable 2. Univariate Association with Death and Current Use of NSAIDs for Each Covariate Included in the Propensity Score

eTable 3. Risks, Risk Differences, and Risk Ratios for Ibuprofen Use, Main Cohort

eTable 4. Estimates of Risk Ratios When Investigating Potential Effect Modification by Age, Sex, and History of Cardiovascular Disease

eTable 5. Risks, Risk Differences, and Risk Ratios for In-Hospital Death, Main Population

eTable 6. Risks, Risk Differences, and Risk Ratios for Main Cohort Using Inverse Probability of Treatment Weights

eTable 7. Baseline Characteristics of Those Currently Using NSAIDs and Those Currently Using Acetaminophen

eTable 8. Risks, Risk Differences, and Risk Ratios for Cohort Diagnosed With any Bacterial Pneumonia

eTable 9. Risks, Risk Differences, and Risk Ratios for Cohort Diagnosed With *Streptococcus pneumoniae* Pneumonia

This supplementary material has been provided by the authors to give readers additional information about their work.

© 2020 Lund LC et al. *JAMA Network Open.*

eAppendix 1. Codes Used to Define Eligibility Criteria, Exposures, Outcomes, and Covariates

Eligibility criteria	*Coding system*	*Code*
Pneumonia or hospitalized influenza	ICD-10	B01.2, J09-J18, A48.1, A70
Bacterial pneumonia	ICD-10	J13-J15, J16.0, J17.0, A48.1, A70
S. pneumoniae pneumonia	ICD-10	J13
Exposures		
NSAID	ATC	M01A (excluding M01AX)
Acetaminophen	ATC	N02BE01
Prescription drug use		
Antihypertensives	ATC	C08, C03CA, C07, C09
Antidiabetic drugs	ATC	A10
Low-dose aspirin	ATC	B01AC06, B01AC30, N02BA01
Immunosuppressants	ATC	L04
Any antibiotic	ATC	J01
Beta-lactamase sensitive penicillins	ATC	J01CE
Extended-spectrum penicllins	ATC	J01CA02, J01CA04
Macrolides	ATC	J01FA
Amoxicillin and clavulanic acid	ATC	J01CR
Fluoroquinolones used for upper respiratory tract infections	ATC	J01MA14
Other antibiotics	ATC	J01 (excluding J01CE, J01CA02, J01CA04, J01FA, J01CR, J01MA14)
Opioids	ATC	N02A
Benzodiazepines	ATC	N05BA, N05CD
Non-benzodiazepine benzodiazepine receptor agonists	ATC	N05CF
1st gen. antipsychotics	ATC	N05AA, N05AB, N05AC, N05AD, N05AF, N05AG, N05AL01

© 2020 Lund LC et al. *JAMA Network Open.*

2nd gen. antipsychotics	ATC	N05AE, N05AH, N05AX, N05AL05
Systemic glucocorticoids	ATC	H02AB
Inhaled corticosteroids	ATC	R03BA01-09, R03AK06-08, R03AK10, R03AK11
History of		
Asthma	ICD-10	J45, J46
Chronic obstructive pulmonary disease	ICD-10	J41-J44
Cardiovascular disease	ICD-10	I20-I25, I47-I50
Ischaemic stroke	ICD-10	I63, I64
Chronic kidney disease	ICD-10	N00, N01, N03-N06, N08.8, N14.1, N14.2, N16.8, N17, N25.1, N26, N27
Liver disease	ICD-10	B18, K70.0- K70.3, K709, K71, K73, K74, K76.0, B15.0, B16.0, B16.2, B19.0, K70.4, K72, K76.6, I85
Alcohol related disorders	ICD-10	F10, E24.4, G31.2, G62.1, G72.1, I42.6, K29.2, K70, K85.2, K86.0, Q86.0, Z50.2, Z71.4, Z72.1
Dementia	ICD-10	F01-F04
Cancer	ICD-10	C00-C97, excluding C44
Human immunodeficiency virus	ICD-10	B20-B22, B24
Obesity	ICD-10	E66
Hemiplegia and paraplegia	ICD-10	G04.1, G11.4, G80.1, G80.2, G83.0-G83.4, G83.9
Outcomes		
Admission to intensive care unit	SKS	NABB, NABE
Pleural empyema or lung abscess	ICD-10	J85, J86

© 2020 Lund LC et al. *JAMA Network Open.*

eAppendix 2. NSAID Sales in Denmark, Used for Calculation of Over-the-Counter NSAID Sales

Source:https://medstat.dk

	DDD sold (x 1000)									
**Drug (ATC code)**	**2010**	**2011**	**2012**	**2013**	**2014**	**2015**	**2016**	**2017**	**2018**	**Total**
NSAIDs excluding glucosamine, overall sale	72.412	73.722	72.556	69.025	65.614	66.389	64.812	60.784	57.663	602.977
Ibuprofen (M01AE01), overall sale	48.110	50.899	52.577	51.474	49.844	51.594	51.550	48.999	46.856	451.903
Ibuprofen (M01AE01), over the counter sale	14.914	16.797	17.876	16.986	10.467	11.351	11.341	11.270	11.714	122.716

© 2020 Lund LC et al. *JAMA Network Open.*

eTable 1. Risk, Risk Differences, and Risk Ratios for Main Cohort, Unmatched Cohorts

**Exposure**	**Risk (exposed), %**	**Risk (unexposed), %**	**Risk difference, %**	**Risk ratio**
Intensive care unit admission				
Current use	20.0 (16.6-23.4)	13.3 (12.5-14.0)	6.7 (3.2-10.3)	1.51 (1.26-1.81)
Incident use	25.0 (13.7-36.3)	13.3 (12.5-14.0)	11.7 (0.4-23.1)	1.89 (1.19-2.98)
Chronic use	28.2 (20.8-35.6)	13.3 (12.5-14.0)	14.9 (7.5-22.4)	2.13 (1.62-2.78)
30-day mortality				
Current use	7.1 (4.9-9.3)	7.8 (7.2-8.4)	-0.7 (-3.0-1.6)	0.91 (0.66-1.26)
Incident use	7.1 (0.4-13.9)	7.8 (7.2-8.4)	-0.6 (-7.4-6.1)	0.92 (0.36-2.37)
Chronic use	7.0 (2.8-11.3)	7.8 (7.2-8.4)	-0.7 (-5.0-3.5)	0.90 (0.49-1.65)

© 2020 Lund LC et al. *JAMA Network Open.*

eTable 2. Univariate Association with Death and Current Use of NSAIDs for Each Covariate Included in the Propensity Score

**Covariate**	**RR(exposure)**	**RR(outcome)**
Prescription drug use		
Antihypertensives	1.19 (0.96-1.46)	1.11 (0.89-1.38)
Antidiabetic drugs	1.11 (0.88-1.39)	0.86 (0.69-1.07)
Low-dose aspirin	0.90 (0.71-1.13)	1.04 (0.86-1.26)
Immunosuppressants	1.23 (0.71-2.15)	0.91 (0.50-1.65)
Recent antibiotics	1.43 (1.18-1.74)	1.54 (1.28-1.85)
Beta-lactams (monotherapy)	1.14 (0.89-1.47)	1.51 (1.21-1.88)
Beta-lactams combined with beta-lactamase inhibitors	1.64 (1.17-2.30)	1.59 (1.15-2.19)
Macrolides	1.48 (1.04-2.09)	1.04 (0.69-1.55)
Quinolones	0.88 (0.35-2.20)	1.56 (0.77-3.17)
Opioids	2.29 (1.90-2.75)	1.45 (1.21-1.73)
Benzodiazepines	1.57 (1.22-2.03)	1.79 (1.41-2.28)
Non-benzodiazepine benzodiazepine receptor agonists	1.25 (0.97-1.61)	1.27 (1.01-1.60)
1st gen. antipsychotics	1.92 (1.26-2.92)	2.82 (1.89-4.19)
2nd generation antipsychotics	1.23 (0.83-1.83)	2.09 (1.47-2.97)
Systemic glucocorticoids	1.59 (1.26-2.00)	1.44 (1.15-1.80)
Inhaled corticosteroids	1.32 (1.06-1.64)	0.95 (0.76-1.20)
Prior diagnoses		
Asthma	1.05 (0.80-1.39)	0.74 (0.53-1.03)
COPD	1.07 (0.86-1.33)	1.44 (1.20-1.74)
Cardiovascular disease	0.74 (0.60-0.91)	1.23 (1.03-1.46)
Stroke	1.09 (0.80-1.47)	1.53 (1.22-1.91)
Chronic kidney disease	0.72 (0.43-1.18)	1.20 (0.78-1.83)
Liver disease	1.01 (0.63-1.62)	2.29 (1.53-3.41)
Alcohol related disorders	1.02 (0.73-1.43)	2.98 (2.27-3.90)
Dementia	0.62 (0.30-1.27)	2.05 (1.46-2.86)
Cancer	1.05 (0.84-1.30)	1.11 (0.92-1.35)
HIV	-	-
Obesity	1.02 (0.75-1.40)	1.01 (0.73-1.41)
Hemiplegia and paraplegia	0.86 (0.38-1.99)	1.15 (0.55-2.40)

© 2020 Lund LC et al. *JAMA Network Open.*

eTable 3. Risks, Risk Differences, and Risk Ratios for Ibuprofen Use, Main Cohort

Exposure	Risk (exposed), %	Risk (unexposed), %	Risk difference, %	Risk ratio
Intensive care unit admission				
Current use	20.0 (16.6-23.4)	16.0 (12.8-19.1)	4.0 (-0.6-8.7)	1.25 (0.96-1.63)
Incident use	25.0 (13.7-36.3)	17.9 (7.8-27.9)	7.1 (-8.0-22.3)	1.40 (0.68-2.88)
Chronic use	28.2 (20.8-35.6)	14.8 (9.0-20.6)	13.4 (4.0-22.8)	1.90 (1.19-3.06)
30-day mortality				
Current use	7.1 (4.9-9.3)	6.9 (4.7-9.1)	0.2 (-2.9-3.3)	1.03 (0.66-1.60)
Incident use	7.1 (0.4-13.9)	7.1 (0.4-13.9)	0.0 (-9.5-9.5)	1.00 (0.26-3.80)
Chronic use	7.0 (2.8-11.3)	4.9 (1.4-8.5)	2.1 (-3.4-7.6)	1.43 (0.56-3.65)

© 2020 Lund LC et al. *JAMA Network Open.*

eTable 4. Estimates of Risk Ratios When Investigating Potential Effect Modification by Age, Sex, and History of Cardiovascular Disease

Effect modifier		ICU admission	p-value	30-day mortality	p-value	In-hospital death	p-value
Age	65+	1.04 (0.68-1.58)		0.98 (0.56-1.71)		0.99 (0.53-1.84)	
	<65	1.46 (1.05-2.04)	0.21	1.07 (0.52-2.21)	0.84	1.16 (0.56-2.41)	0.75
Sex	Male	0.89 (0.60-1.31)		0.86 (0.46-1.64)		0.96 (0.49-1.90)	
	Female	1.67 (1.16-2.41)	0.02	1.21 (0.65-2.24)	0.46	1.18 (0.60-2.29)	0.68
Cardiovascular disease	Yes	0.84 (0.51-1.39)		0.61 (0.28-1.30)		0.50 (0.19-1.31)	
	No	1.45 (1.07-1.98)	0.07	1.33 (0.77-2.31)	0.10	1.40 (0.80-2.45)	0.06

© 2020 Lund LC et al. *JAMA Network Open.*

eTable 5. Risks, Risk Differences, and Risk Ratios for In-Hospital Death, Main Population

Exposure	Risk (exposed), %	Risk (unexposed), %	Risk difference, %	Risk ratio
Current use	6.3 (4.3-8.4)	6.0 (3.9-8.0)	0.4 (-2.5-3.3)	1.06 (0.66-1.71)
Incident use	7.1 (0.4-13.9)	7.1 (0.4-13.9)	0.0 (-9.5-9.5)	1.00 (0.26-3.80)
Chronic use	7.0 (2.8-11.3)	4.2 (0.9-7.5)	2.8 (-2.5-8.2)	1.67 (0.62-4.46)

© 2020 Lund LC et al. *JAMA Network Open.*

eTable 6. Risks, Risk Differences, and Risk Ratios for Main Cohort Using Inverse Probability of Treatment Weights

Exposure	Risk (exposed), %	Risk (unexposed), %	Risk difference, %	Risk ratio
Intensive care unit admission				
Current use	15.1 (11.7-18.4)	13.4 (12.6-14.2)	1.7 (-1.7-5.1)	1.15 (0.88-1.51)
Incident use	17.5 (5.5-29.6)	13.5 (12.7-14.3)	4.1 (-7.9-16.0)	1.36 (0.60-3.13)
Chronic use	17.5 (8.4-26.5)	13.6 (12.7-14.4)	3.9 (-5.1-13.0)	1.35 (0.72-2.53)
30-day mortality				
Current use	7.4 (4.3-10.5)	7.8 (7.1-8.4)	-0.4 (-3.5-2.8)	0.95 (0.60-1.50)
Incident use	5.6 (-1.2-12.4)	7.4 (6.8-8.0)	-1.8 (-8.6-4.9)	0.74 (0.20-2.67)
Chronic use	12.8 (0.5-25.2)	7.4 (6.8-8.0)	5.4 (-6.9-17.7)	1.84 (0.61-5.54)

© 2020 Lund LC et al. *JAMA Network Open.*

eTable 7. Baseline Characteristics of Those Currently Using NSAIDs and Those Currently Using Acetaminophen

	**Unmatched**			**Matched**		
	NSAID users (n=520)	Acetaminophen users (n=1,786)	SMD	NSAID users (n=484)	Acetaminophen users (n=484)	SMD
Age, median (IQR)	66 (54-74)	76 (67-83)	0.74	68 (56-75)	65 (55-76)	0.02
Sex (male)	232 (44.6)	738 (41.3)	0.07	212 (43.8)	218 (45.0)	0.02
Current drug use						
Antihypertensives	363 (69.8)	1,508 (84.4)	0.35	351 (72.5)	344 (71.1)	0.03
Antidiabetic drugs	102 (19.6)	435 (24.4)	0.11	97 (20.0)	98 (20.2)	0.01
Low-dose aspirin	101 (19.4)	522 (29.2)	0.23	100 (20.7)	104 (21.5)	0.02
Immunosuppressants	14 (2.7)	55 (3.1)	0.02	14 (2.9)	18 (3.7)	0.05
Recent antibiotics	159 (30.6)	533 (29.8)	0.02	151 (31.2)	153 (31.6)	0.01
Beta-lactams (monotherapy)	78 (15.0)	286 (16.0)	0.03	75 (15.5)	71 (14.7)	0.02
Beta-lactams combined with beta-lactamase inhibitors	41 (7.9)	122 (6.8)	0.04	40 (8.3)	41 (8.5)	0.01
Macrolides	39 (7.5)	105 (5.9)	0.06	36 (7.4)	37 (7.6)	0.01
Quinolones	5 (1.0)	26 (1.5)	0.05	NA	NA	NA
Opioids	232 (44.6)	926 (51.8)	0.15	220 (45.5)	218 (45.0)	0.01
Non-benzodiazepine benzodiazepine receptor agonists	80 (15.4)	292 (16.3)	0.03	77 (15.9)	73 (15.1)	0.02
Benzodiazepines	79 (15.2)	342 (19.1)	0.11	77 (15.9)	73 (15.1)	0.02
1st gen. antipsychotics	27 (5.2)	49 (2.7)	0.13	23 (4.8)	19 (3.9)	0.04
2nd gen. antipsychotics	29 (5.6)	77 (4.3)	0.06	26 (5.4)	20 (4.1)	0.06
Systemic glucocorticoids	98 (18.8)	347 (19.4)	0.01	92 (19.0)	98 (20.2)	0.03
Inhaled corticosteroids	115 (22.1)	398 (22.3)	0.00	109 (22.5)	108 (22.3)	0.00

© 2020 Lund LC et al. *JAMA Network Open.*

History of:						
Asthma	64 (12.3)	234 (13.1)	0.02	62 (12.8)	56 (11.6)	0.04
COPD	113 (21.7)	556 (31.1)	0.21	111 (22.9)	111 (22.9)	0.00
Cardiovascular disease	146 (28.1)	923 (51.7)	0.50	143 (29.5)	145 (30.0)	0.01
Ischaemic stroke	51 (9.8)	279 (15.6)	0.18	51 (10.5)	56 (11.6)	0.03
Chronic kidney failure	17 (3.3)	93 (5.2)	0.10	16 (3.3)	18 (3.7)	0.02
Liver disease	20 (3.8)	59 (3.3)	0.03	19 (3.9)	16 (3.3)	0.03
Alcohol related disorders	40 (7.7)	149 (8.3)	0.02	39 (8.1)	30 (6.2)	0.07
Dementia	8 (1.5)	84 (4.7)	0.18	8 (1.7)	(n<5)	NA
Cancer	117 (22.5)	455 (25.5)	0.07	113 (23.3)	116 (24.0)	0.01
HIV	NA	NA	NA	NA	NA	NA
Obesity	47 (9.0)	193 (10.8)	0.06	47 (9.7)	49 (10.1)	0.01
Hemiplegia and paraplegia	6 (1.2)	44 (2.5)	0.10	6 (1.2)	8 (1.7)	0.03

© 2020 Lund LC et al. *JAMA Network Open.*

eTable 8. Risks, Risk Differences, and Risk Ratios for Cohort Diagnosed With any Bacterial Pneumonia

Exposure	Risk (exposed), %	Risk (unexposed), %	Risk difference, %	Risk ratio
Intensive care unit admission				
Current use	14.3 (13.5-15.0)	12.5 (11.7-13.2)	1.8 (0.8-2.9)	1.15 (1.06-1.24)
Incident use	16.5 (14.3-18.6)	11.5 (9.7-13.4)	4.9 (2.1-7.8)	1.43 (1.16-1.76)
Chronic use	16.5 (15.0-18.1)	13.3 (11.9-14.7)	3.2 (1.1-5.3)	1.24 (1.08-1.43)
Pleuropulmonary complications				
Current use	2.1 (1.8-2.4)	1.0 (0.8-1.2)	1.1 (0.7-1.5)	2.13 (1.63-2.77)
Incident use	3.9 (2.8-5.0)	1.1 (0.5-1.7)	2.8 (1.5-4.1)	3.67 (1.95-6.91)
Chronic use	2.0 (1.4-2.6)	1.1 (0.6-1.5)	0.9 (0.2-1.6)	1.87 (1.13-3.09)
30-day mortality				
Current use	13.9 (13.2-14.7)	14.3 (13.6-15.1)	-0.4 (-1.5-0.7)	0.97 (0.90-1.05)
Incident use	14.8 (12.7-16.9)	11.4 (9.6-13.3)	3.3 (0.6-6.1)	1.29 (1.04-1.60)
Chronic use	12.7 (11.3-14.1)	12.8 (11.4-14.2)	-0.0 (-2.0-1.9)	1.00 (0.85-1.16)

© 2020 Lund LC et al. *JAMA Network Open.*

eTable 9. Risks, Risk Differences, and Risk Ratios for for Cohort Diagnosed With *Streptococcus pneumoniae* Pneumonia

Exposure	Risk (exposed), %	Risk (unexposed), %	Risk difference, %	Risk ratio
Intensive care unit admission				
Current use	25.4 (21.4-29.4)	20.6 (16.9-24.3)	4.8 (-0.6-10.3)	1.23 (0.97-1.57)
Incident use	27.6 (17.6-37.7)	14.5 (6.6-22.4)	13.2 (0.4-26.0)	1.91 (0.99-3.68)
Chronic use	26.4 (19.6-33.3)	15.7 (10.1-21.4)	10.7 (1.8-19.6)	1.68 (1.08-2.62)
Pleuropulmonary complications				
Current use	6.6 (4.3-8.9)	2.4 (1.0-3.8)	4.2 (1.5-6.8)	2.73 (1.38-5.38)
Incident use	9.2 (2.7-15.7)	3.9 (-0.4-8.3)	5.3 (-2.6-13.1)	2.33 (0.63-8.69)
Chronic use	8.2 (3.9-12.4)	1.9 (-0.2-4.0)	6.3 (1.5-11.0)	4.33 (1.26-14.91)
30-day mortality				
Current use	9.0 (6.4-11.6)	7.0 (4.7-9.4)	2.0 (-1.5-5.5)	1.28 (0.82-2.00)
Incident use	6.6 (1.0-12.2)	9.2 (2.7-15.7)	-2.6 (-11.2-5.9)	0.71 (0.24-2.15)
Chronic use	11.3 (6.4-16.2)	6.9 (3.0-10.9)	4.4 (-1.9-10.7)	1.64 (0.80-3.35)

© 2020 Lund LC et al. *JAMA Network Open.*
